# Effects of Adiponectin on Diastolic Function in Mice Underwent Transverse Aorta Constriction

**DOI:** 10.1007/s12265-019-09913-1

**Published:** 2019-10-16

**Authors:** Xueting Han, Yanyan Wang, Mingqiang Fu, Yu Song, Jingfeng Wang, Xiaotong Cui, Yuyuan Fan, Juan Cao, Jie Luo, Aijun Sun, Yunzeng Zou, Kai Hu, Jingmin Zhou, Junbo Ge

**Affiliations:** 1 Department of Cardiology, Shanghai Institute of Cardiovascular Diseases, Zhongshan Hospital, Fudan University, Shanghai, China; 2 North Sichuan Medical College, Nanchong, Sichuan China

**Keywords:** Diastolic dysfunction, Transverse aorta constriction, Adiponectin, Titin, AMPK

## Abstract

Diastolic dysfunction is common in various cardiovascular diseases, which could be affected by adiponectin (APN). Nevertheless, the effects of APN on diastolic dysfunction in pressure overload model induced by transverse aorta constriction (TAC) remain to be further elucidated. Here, we demonstrated that treatment of APN attenuated diastolic dysfunction and cardiac hypertrophy in TAC mice. Notably, APN also improved active relaxation of adult cardiomyocytes, increased N2BA/N2B ratios of titin isoform, and reduced collagen type I to type III ratio and lysyl oxidase (Lox) expressions in the myocardial tissue. Moreover, APN supplementation suppressed TAC-induced oxidative stress. In vitro, inhibition of AMPK by compound C (Cpc) abrogated the effect of APN on modulation of titin isoform shift and the anti-hypertrophic effect of APN on cardiomyocytes induced by AngII. In summary, our findings indicate that APN could attenuate diastolic dysfunction in TAC mice, which are at least partially mediated by AMPK pathway.

**Electronic supplementary material:**

The online version of this article (10.1007/s12265-019-09913-1) contains supplementary material, which is available to authorized users.

## Introduction

Left ventricular (LV) diastolic dysfunction is a key defining feature of various cardiovascular diseases [1]. It is reported that, among the patient diagnosed with heart failure (HF) each year, approximately 50% of them showed diastolic dysfunction [2]. Diastolic dysfunction has been observed by echocardiography in two-thirds of patients at rest [3, 4]. Many others displayed elevated LV filling pressures during the stress of exercise, indicating an earlier stage of LV diastolic dysfunction [5]. Impaired active myocardial relaxation and increased passive stiffness are the two major elements of LV diastolic dysfunction, which jointly lead to elevated LV filling pressure [6]. Passive stiffness is determined by the extracellular matrix and cardiomyocytes. In extracellular level, myocardial stiffness is largely determined by the total amount of collagen, abundance of collagen type I, and degree of collagen cross-linking [4]. Collagen cross-linking is further manipulated by the enzyme lysyl oxidase (Lox) in mechanical active hearts [7]. In cardiomyocyte level, modifications of titin compliance via isotype switching and post-translational changes and are crucial for myocardial stiffness status [4, 8, 9]. Clinical studies showed that diastolic dysfunction is an independent risk factor in patents with various cardiovascular diseases including hypertensive patients with left ventricular hypertrophy.

Emerging evidence indicates that adiponectin (APN), an adipokine mainly deliver from white adipose tissue, plays an important role in the pathology of diastolic dysfunction. Clinical trials found that APN levels were lower in obese individuals [10] and women with cardiac diastolic dysfunction [11]. In ischemia/reperfusion-induced mice, APN could reduce left ventricular end diastolic filling pressure by increasing NO production via phosphorylation of eNOS at Ser1177 and inhibition of iNOs expression [12, 13]. APN knock-out mice underwent aldosterone infusion displayed aggravated diastolic function, which can be attenuated by APN through phosphorylation of calcium handling protein phospholamban (PLB) [14]. Collectively, these findings indicate that APN exerts positive effects on diastolic function developed from difference causes. However, the effects of APN on pathological mechanisms of diastolic dysfunction in active relaxation and passive stiffness and the related molecular signaling pathway remain to be elucidated.

In this study, we utilized a mouse model induced by transverse aortic constriction (TAC), which resembles aspects of left ventricular hypertrophy (LVH) and diastolic dysfunction [15–17]. By intraperitoneal injection with APN, we aimed to investigate the role of APN on TAC-induced cardiac diastolic dysfunctions and clarify the potent molecular mechanisms.

## Methods

### Animal Model of Diastolic Dysfunction Induced by TAC

Male C57BL/6J mice (8 weeks old, weighing 20–22 g) were purchased from Shanghai SLAC Laboratory Animal Co., Ltd. TAC was performed as described [18]. In brief, mice were anesthetized by intraperitoneal injection with 50 mg/kg sodium pentobarbital. The transverse aorta was constricted between the origins of the innominate artery and the left common carotid artery with a 5–0 silk braided non-absorbable suture by ligating the aorta together with a blunted 27-gauge needle, which was removed later; a constriction of 0.4 mm in diameter was the product of this endeavor. To evaluate the mouse model of diastolic dysfunction, mice after 2 and 4 weeks of TAC were studied via echocardiography. Echocardiographic data for mice after 2 weeks of TAC are given in Online Resource 1(Table S1, Fig. S1).

### Animal Treatment

Male C57BL/6J mice (8 weeks old, weighing 20–22 g) were divided into 3 groups: (1) sham; (2) TAC; and (3) TAC+APN (*n* = 12 mice for each group). Mice in sham group underwent similar surgical procedure without constriction of the aorta and received intraperitoneally injection of physiological saline beginning on the day after 2 weeks of sham surgery (1 time per day for 2 weeks). Mice in TAC group received equal volume of saline injection 2 weeks after TAC (1 time per day for 2 weeks). Mice in TAC+APN group were intraperitoneally injected with APN (Biovision, SFO, USA) (0.25 μg g^−1^ d^−1^) [19] 2 weeks after TAC (1 time per day for 2 weeks). Echocardiography was performed at the end of 4 weeks post-TAC to assess cardiac structure and function.

### Echocardiography

Mice were anesthetized with an isoflurane face mask with a concentration of 1.5–2%. Heart rate during echocardiographic study was maintained 350 to 400 bpm for Doppler studies for the separation of E and A velocities. Transthoracic echo images were obtained with a Vevo 2100 High-Resolution Imaging System (Visual-Sonics). Images were acquired using a high-resolution (30 MHz) transducer. Fractional shortening (FS), left ventricular ejection fraction (LVEF), ventricular dimensions, and volumes were obtained via a M-mode. Passive left ventricular filling peak velocity (*E*, mm/s) and atrial contraction flow peak velocity (*A*, mm/s) were obtained by mitral valve Doppler flow. Early (*E*’, mm/s) and late (*A*′, mm/s) diastolic mitral annular motion velocity were obtained by tissue Doppler imaging from the apical 4 chamber view.

### Adult Cardiac Myocytes Isolation

Four weeks after surgery, ventricular myocytes from all groups were isolated via a simplified Langendorff-free method as described [20]. Details are available at Supplemental Methods in Online Resource 1.

### Assessment of Cardiomyocyte Relaxation

Cells were then perfused with a standard Tyrode’s solution. Mechanical properties of cells were assessed using an IonOptix™ soft-edge system. Cells were placed in a chamber mounted on the stage of an Olympus IX-70 microscope and field-stimulated at 1-Hz frequency by MyoPacer stimulator (IonOptix Co, MA, USA). Cell mechanics were assessed by the indicated indices: resting cell length, peak shortening, maximal velocities of shortening/re-lengthening (±dL/dt), time-to-50% re-lengthening (TR50), time-to-90% re-lengthening (TR90) [21]. All measurements were analyzed by IonWizard 6.3 software.

### Morphological and Histological Analysis

All mice were weighed and then euthanized via neck dislocation while under deep anesthesia. Hearts were rapidly excised and rinsed with cold physiological saline. After absorbing physiological saline with clean filter paper, we recorded mice heart weigh, body weight, and tibia length. The heart to body weight ratios (HW/BW, mg/g) and heart weight to tibia length ratios (HW/TL, mg/mm) were calculated. Mid-ventricular cross-sections of mice hearts were excised and fixed in 4% paraformaldehyde for 24 h. The rest were flash frozen in liquid nitrogen and stored at − 80 °C for further analysis. Fixed samples were then dehydrated through a graded series of ethanol, diaphonized with Xylol and embedded in paraffin. For histological analysis, samples were transversely sectioned (5-μm thick) and mounted on glass slides. Five sections per heart were stained with hematoxylin and eosin (HE) and Masson’s Trichrome. Slides were examined and analyzed to determine the cross-sectional area and fibrotic area. All measurements were conducted using NIH Image J (1.51e) software.

### Western Blot Analysis

Proteins of LV samples or cardiomyocytes were extracted by homogenizing samples in lysis buffer. Sample was loaded on SDS-PAGE gels and was then transferred to polyvinylidene fluoride (PVDF) membranes (Millipore, MA, USA). After being blocked with 5% bovine serum albumin (BSA), membranes were incubated with primary antibodies against AMPKα (Cell Signaling Technology, MA, USA), p-AMPK (Thr172) (Cell Signaling Technology, MA, USA), collagen type I (Santa Cruz Biotechnology, TX, USA), collagen type III (Santa Cruz Biotechnology, TX, USA), Lox (abcam, Cambridge, US), and MnSOD (abcam, Cambridge, US) overnight at 4 °C. Membranes were washed by TBST and further incubated with appropriate horseradish peroxidase-conjugated secondary antibodies (Abbkine, CA, USA) at 37 °C for 2 h. Finally, protein blots were visualized by ECL Plus (Thermo Fisher Scientific, MA, USA) and the relative expression levels were normalized to a loading control of GAPDH (Cell Signaling Technology, MA, USA).

### Total RNA Extraction and qPCR

Total RNAs were extracted using the TRIzol reagent (Invitrogen, CA, USA). qPCR was performed on a CFX Connect ™ Real-Time System (Bio-Rad Laboratories, Inc., CA, USA) with SYBR® Premix Ex Taq™ Kit. The primer sequences are listed in Table 1. The relative quantifications of mRNA levels were calculated with the software of the PCR system by standard 2^−▵▵Ct^ relative quantification method.

**Table 1:** Primer sequences used in the present study

Genes	Primer sequence
mouse-ANF	forward-AAGAACCTGCTAGACCACCTGGAG
	reverse-TGCTTCCTCAGTCTGCTCACTCAG
mouse-BNP	forward-GGAAGTCCTAGCCAGTCTCCAGAG
	reverse-GCCTTGGTCCTTCAAGAGCTGTC
mouse-p^22phox^	forward-CGTGGCTACTGCTGGACGTT
	reverse-GCACACCTGCAGCGATAGAG
mouse-gp^91phox^	forward-AGCTATGAGGTGGTGATGTTAGTGG
	reverse-CACAATATTTGTACCAGACAGACTTGAG
mouse-p^67phox^	forward-TGGACTTCGGATTCACCCTCAGTC
	reverse-CACCTTGAGCATGTAAGGCATAGG
mouse-Rac1	forward-CCCCACCGTCTTTGACAACT
	reverse-CATAGGCCCAGATTCACTGGTT
mouse-p^47phox^	forward-TTCCATCCCCAAATGCAAAG
	reverse-TCAGATGCCCTAAAACCGGAG
rat-ANF	forward-GAAGATGCCGGTAGAAGATGAG
	reverse-AGAGCCCTCAGTTTGCTTTTC
rat-BNP	forward-GGTGCTGCCCCAGATGATT
	reverse-CTGGAGACTGGCTAGGACTTC

### Titin Isoform Separation and Identification

Flash-frozen LV tissue was prepared as previously described [22–24]. Details are shown in Supplemental Methods in Online Resource 1. For the identification of titin, nano-HPLC-MS/MS analysis was performed. All MS/MS samples were analyzed using Mascot to search the Swissprot Mouse database. Details and additional data are given in the Online Resource 1 (Supplemental Methods and Fig. S2) and Online Resource 2.

### Neonatal Rat Cardiac Myocytes Isolation and Treatment

Neonatal rat cardiac myocytes (NRCMs) were isolated by enzymatic digestion and cultured as previously described [25]. The isolated myocytes were cultured in Dulbecco Modified Eagle medium (DMEM) containing 10% fetal bovine serum (FBS) to ensure cell adhesion. Before each experiment, cells were placed in serum-free Dulbecco Modified Eagle medium (DMEM) for 24 h. For titin isoform studies, cells were treated with AngII (1 μmol/L) [26] for 5 days in the presence or absence of APN (30 μg/mL) [25] or Cpc (6 μmol/L). For gene studies, cells were treated with AngII for 12 h. APN or Cpc (AMPK inhibitor) was added 1 h prior to AngII stimulation. For the examination of cardiac myocyte size, cells were seeded in a 24-well plate. After indicated treatment for 48 h, cell immunofluorescence analysis was performed.

### Immunofluorescence Analysis

After indicated treatment, cardiomyocytes were rinsed with PBS for 3 times and fixed with 4% paraformaldehyde for 20 min. Then, cells were rinsed with PBS and permeabilized with 0.1% Triton X-100 (Merck, Darmstadt, GER) for 5 min; After rinsed with PBS, cells were then stained with α-sarcomeric actin (abcam, Cambridge, UK) followed by a fluorescent secondary antibody (abcam, Cambridge, UK). Cells were visualized under a Leica fluorescence microscope and cell sizes were measured by NIH Image J (1.51e) software.

### Statistical Analysis

All data were analyzed using SPSS 24.0. Normality of distributions was verified by Kolmogorov–Smirnov test. Homoscedasticity was determined by the Levene test. For normal distribution variables with equal variances, differences among 3 groups were determined by one-way ANOVA with Bonferroni post hoc test. Student *t* test was used for two-group comparisons. For non-normal distribution variables or with unequal variances, Kruskal–Wallis test followed by the Dunn post hoc test was used. *P* < 0.05 was considered statistically significant. All data were expressed as the mean ± SEM.

## Results

### Survival

There were 5 in 30 mice died 1 week after TAC and 0 in 12 mice died after sham operation. We began to inject the mice with APN or physiological saline after 2 weeks of surgery. No mice died within each group thereafter.

### General Characteristics

Characteristics of sham-, TAC-, and APN-treated mice are summarized in Table 2. Heart rates were comparable among sham-, TAC-, and APN-treated mice. Heart weight of TAC mice was significantly increased, which were reduced by APN treatment. Accordingly, heart weight to body weight ratios (HW/BW) and heart weight to tibia length ratios (HW/TL) were significantly higher in TAC mice than those in sham mice, and were reduced by the treatment of APN.

**Table 2:** Characteristics of sham-, TAC-, and APN-treated mice

At 4 weeks after surgery	Sham	TAC	TAC+APN
General characteristics			
HR (bpm)	437.81 ± 14.21	438.51 ± 11.11	475.41 ± 13.81
HW (mg)	135 ± 5	195 ± 13**	155 ± 5*^#^
BW (g)	27.22 ± 0.51	27.34 ± 0.55	26.81 ± 0.55
TL (mm)	17.58 ± 0.20	17.75 ± 0.13	17.31 ± 0.16
HW/BW (mg/g)	4.88 ± 0.10	8.11 ± 0.69**	6.20 ± 0.40*^#^
HW/TL (mg/mm)	7.85 ± 0.28	12.86 ± 1.21**	9.80 ± 0.34*^#^

Values represent the mean ± SEM, *n* = 10 per group. **P < 0.01 vs. sham; *P < 0.05 vs. sham; ^##^*P* < 0.01 vs. TAC group; ^#^*P* < 0.05 vs. TAC group

### Echocardiographic Examination

**Fig. 1:**
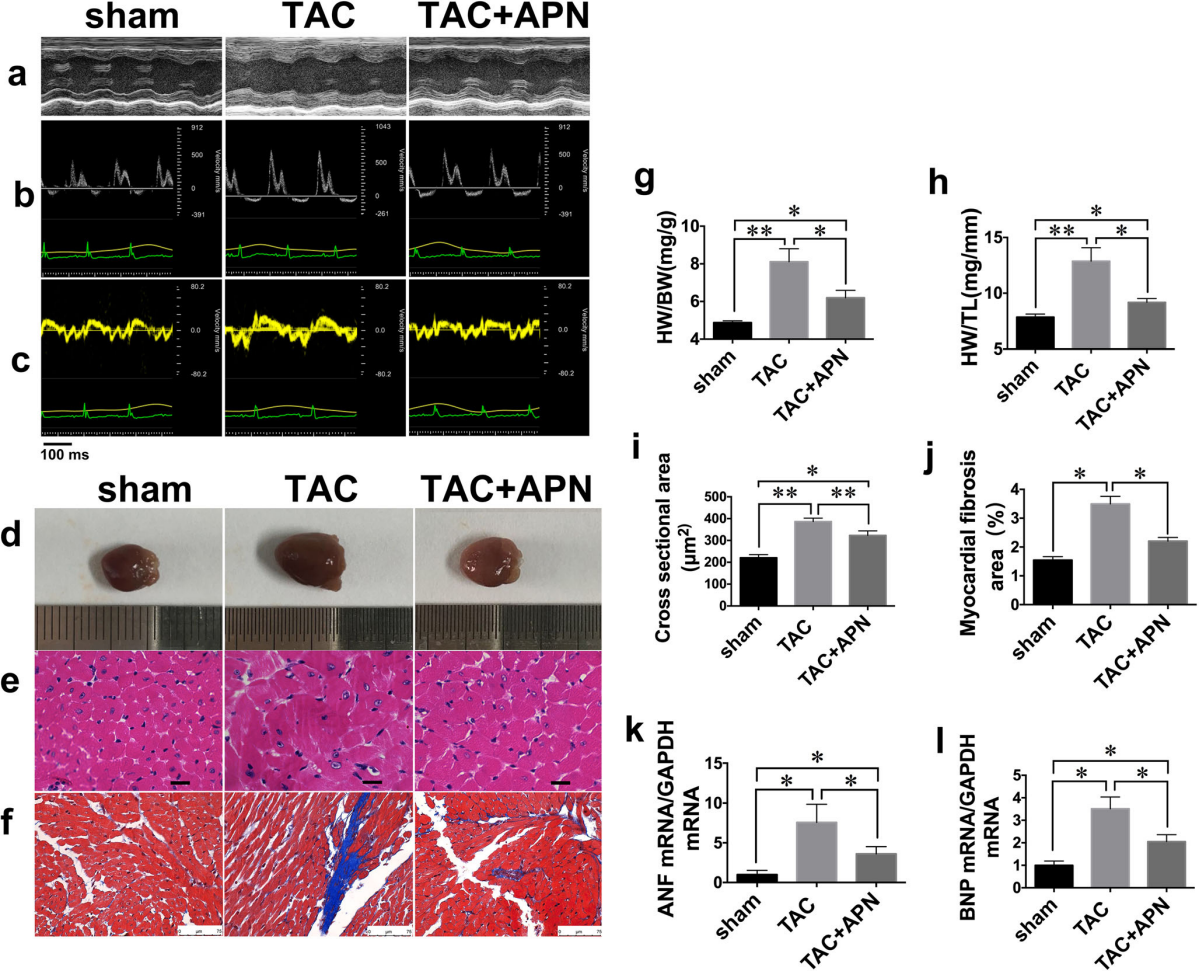
Evaluation of cardiac function, structure, and morphology in three groups of mice. **a** M-mode echocardiography showed increased left ventricular posterior wall thickness (LVPW) in TAC mice while APN-treated mice demonstrated reduced LVPW. **b** Mitral inflow pattern and **c** mitral annular velocity of TAC group revealed progressive diastolic dysfunction while APN-treated mice showed recovery of diastolic dysfunction, *n* = 10 per group. **d** Representative global heart photographs. **e** Hematoxylin and eosin-stained LV transverse sections (original magnification × 400; sale bar, 20 μm). **f** Representative microscopic images of Masson’s trichrome staining (original magnification × 400; sale bar, 75 μm). **g** Quantitative analysis of heart weight to body weight ratio. **h** Quantitative analysis of heart weight to tibia length ratio. **i** Quantitative analysis of cross-sectional area (CSA). **j** Quantification of myocardial fibrotic area (%). **k**, **l** Expressions of hypertrophy-associated genes. GAPDH served as the internal control. ANF, natriuretic peptide A. BNP, natriuretic peptide B. Values represent the mean ± SEM, *n* = 6 per group. **P < 0.01; *P < 0.05

Echocardiographic parameters are summarized in Table 3. TAC mice displayed increased posterior wall thickness (LVPW), compared with sham mice, which were significantly ameliorated by the treatment of APN (Fig.1a). To measure diastolic function, characteristic flow profile of the mitral valve Doppler and tissue Doppler flow was analyzed in apical four-chamber view (Fig. 1b, c). TAC mice exhibited increased *E* velocities, *E*/*A* ratios, *E*/*E*′ ratios and decreased *E*′ velocities, when compared with sham mice. These abnormalities, which indicate impaired LV compliance and increased LV filling pressure, were improved in APN-treated mice. Early filling deceleration time (DT) and isovolumic relaxation time (IVRT) were similar among the three groups. Systolic function of TAC mice was fully preserved, as LVEF and FS were comparable between three groups of mice.

**Table 3:** Echocardiographic parameters of sham-, TAC-, and APN-treated mice

At 4 weeks after surgery	Sham	TAC	TAC+APN
LV structure			
LVPW, d (mm)	0.73 ± 0.04	1.35 ± 0.11*	1.02 ± 0.07*^#^
LVPW, s (mm)	0.78 ± 0.07	1.44 ± 0.09**	1.07 ± 0.05*^#^
LVID, d (mm)	3.79 ± 0.09	3.58 ± 0.07	3.79 ± 0.14
LVID, s (mm)	2.85 ± 0.11	2.63 ± 0.11	2.72 ± 0.23
Diastolic function			
*E* (mm/s)	502.00 ± 12.90	632.00 ± 23.80**	547.20 ± 17.64*^#^
*A* (mm/s)	339.40 ± 14.76	347.50 ± 21.21	338.60 ± 16.69
*E*/*A*	1.49 ± 0.03	1.82 ± 0.05**	1.66 ± 0.03**^#^
*E*′ (mm/s)	21.65 ± 1.82	16 ± 1.24**	20.68 ± 1.09^#^
*E/E*′	23.70 ± 1.52	40.82 ± 1.18**	29.73 ± 0.68**^##^
IVRT (ms)	18.34 ± 1.68	17.10 ± 1.98	19.84 ± 2.04
DT (ms)	22.25 ± 1.82	19.96 ± 3.18	21.96 ± 2.32
Systolic function			
LVEF (%)	57.10 ± 4.52	62.57 ± 3.69	62.47 ± 4.97
FS (%)	31.80 ± 2.98	34.11 ± 2.56	34.16 ± 3.69

Values represent the mean ± SEM, *n* = 10 per group. **P < 0.01 vs. sham; *P < 0.05 vs. sham; ^##^*P* < 0.01 vs. TAC group; ^#^*P* < 0.05 vs. TAC group

### Myocardial Hypertrophy and Fibrosis

TAC significantly increased heart size and cardiomyocyte cross-sectional areas compared with sham mice (Fig. 1e, i). Consistently, mRNA expressions of ANF and BNP, markers of cardiac hypertrophy, were also increased in myocardium of TAC mice compared with those in sham mice (Fig. 1k, l). By the treatment of APN, both cardiomyocytes cross-sectional areas and mRNA expressions of hypertrophic genes were significantly abated. Fibrotic areas in myocardium of TAC mice were increased compared with that of sham mice (Fig. 1f, j), which were reduced in APN-treated mice.

### Cardiomyocyte Mechanics In Vitro

**Fig. 2:**
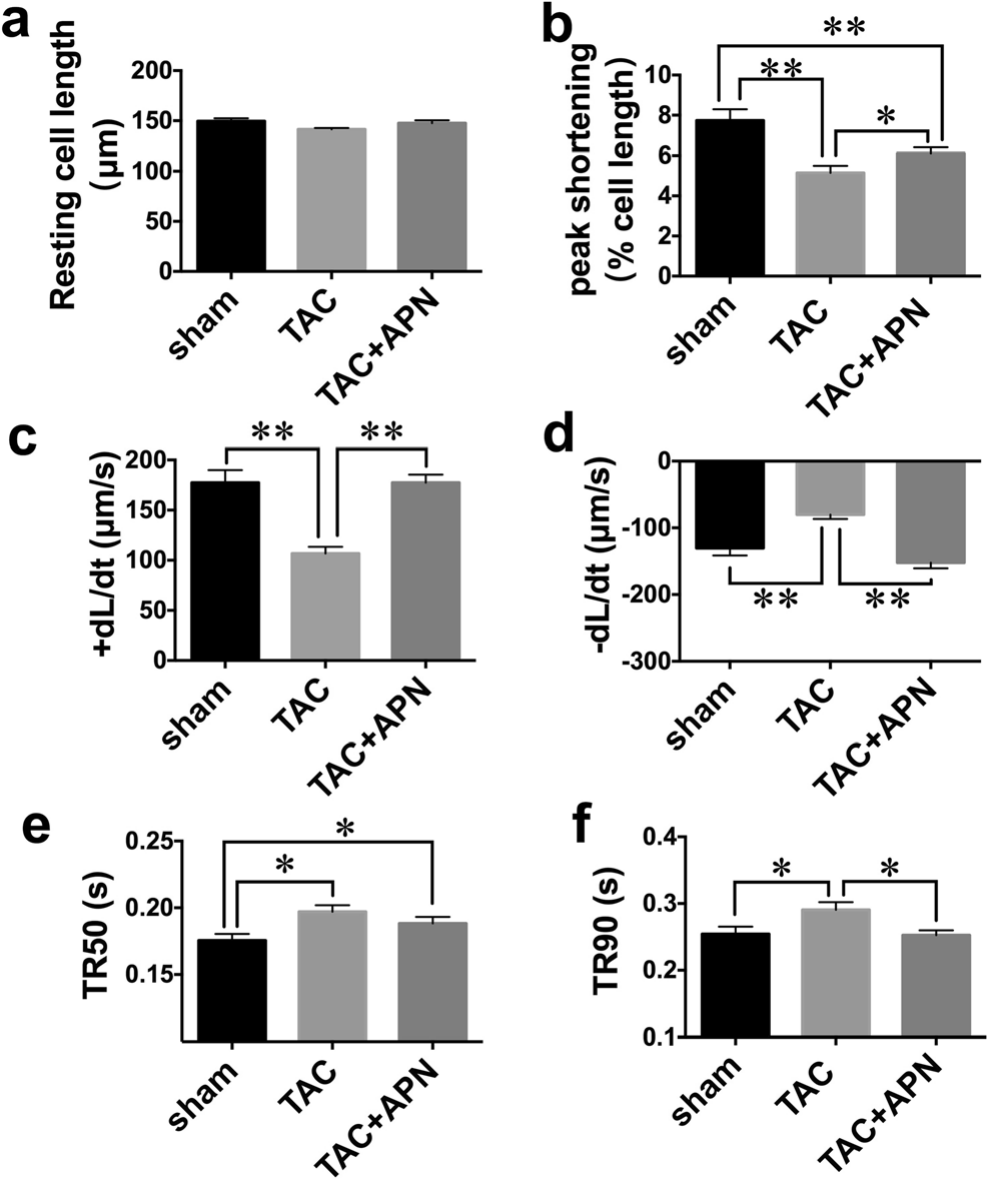
APN improved active relaxation of single myocytes in TAC mice. **a** Resting cell length. **b** Peak shortening. **c** Maximal velocity of shortening (+dL/dt). **d** Maximal velocity of re-lengthening (−dL/dt). **e** Time-to-50% re-lengthening. **f** Time-to-90% re-lengthening. Values represent the mean ± SEM, *n* = 45–50 cells from 5 mice per group. **P < 0.01; *P < 0.05

The source of diastolic dysfunction might be originated within the myocytes. To further investigate myocardial relaxation on a cellular level and the effect of APN on it, we evaluated cardiac mechanics using adult cardiomyocytes isolated from sham-, TAC-, and APN-treated mice. Rest cell lengths were similar among three groups of mice (Fig. 2a). Compared with sham mice, cardiomyocytes of TAC mice manifested decreased peak shortening, maximal rate of cell shortening (+dL/dt), and maximal rate of re-lengthening (−dL/dt) (Fig. 2b, c, d), as well as a significantly prolonged time-to-50% and 90% re-lengthening (TR50, TR90) (Fig. 2e, f). APN treatment restored peak shortening, maximal rate of cell shortening, and re-lengthening. In addition, APN treatment shortened time-to-90% re-lengthening, and time-to-50% re-lengthening was similar between cells from TAC- and APN-treated mice.

### Titin Isoform Shift by APN

**Fig. 3:**
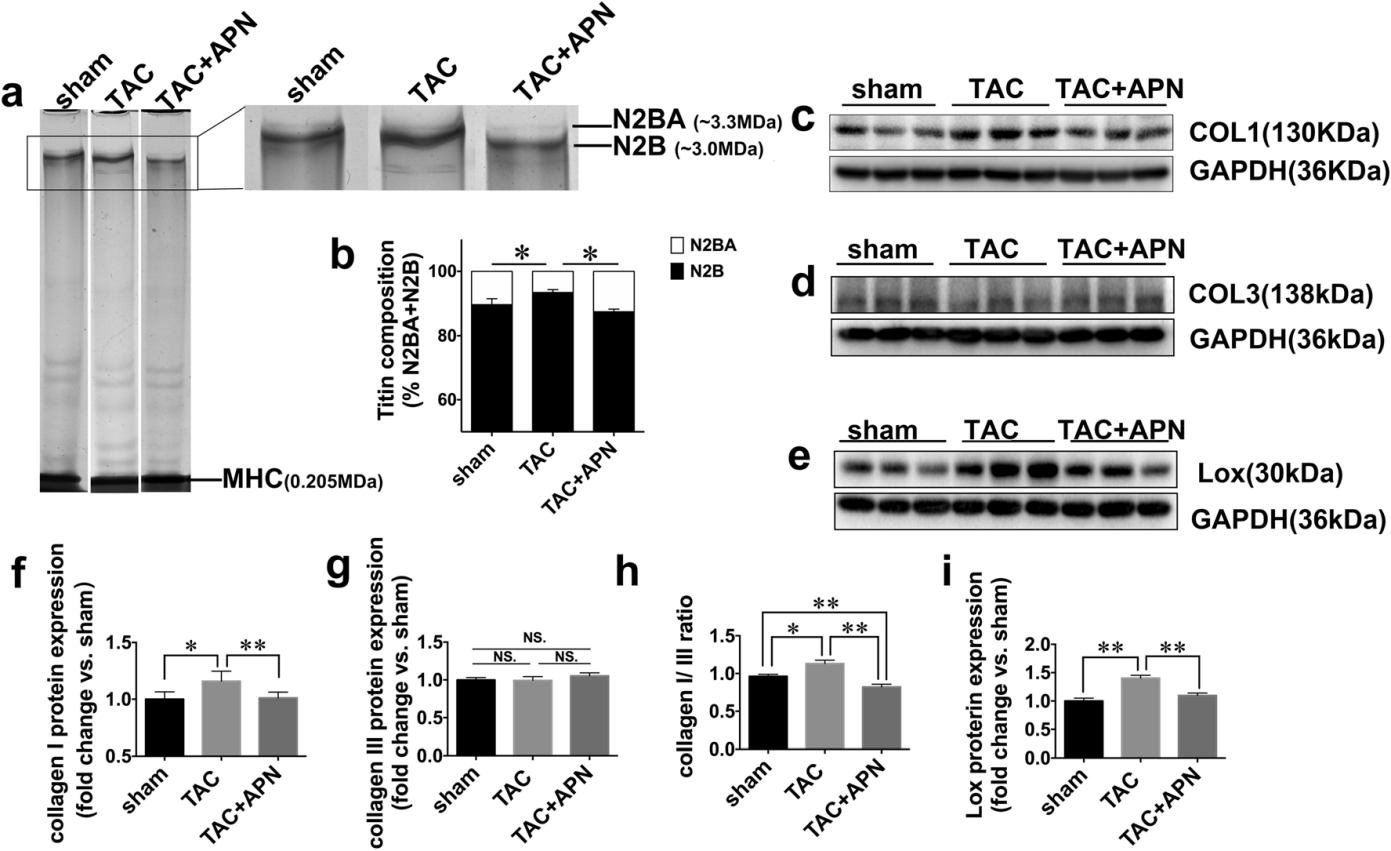
Titin isoform shift and extent of matrix extracellular reorganization in the left ventricular myocardium from three groups of mice. **a** Representative images of titin isoforms in mice LV from indicated groups. **b** Quantitative analysis of titin isoforms. **c–e** Representative blots of collagen type I, collagen type III, and Lox. **f–i** Quantitative analysis of collagen type I, collagen type III, collagen type I to III ratio, and Lox. Values represent the mean ± SEM, *n* = 6 per group. **P < 0.01; *P < 0.05

Titin is a giant sarcomeric protein with two dominant isoforms resulted from alternative splicing: N2BA and N2B. N2BA is larger in size (~ 3.3 MDa) and is a more compliant isoform as compared with N2B (~ 3.0 MDa). In the present study, we sought to investigate the effect of APN on titin isoform slicing. For the identification of titin protein, we extracted proteins from mice myocardium of left ventricle and separated them by 2% agarose strengthened SDS-PAGE gel electrophoresis. Protein band on the gel was analyzed by nano-HPLC-MS/MS and Mascot. As is shown in Online Resource 1(Fig. S2) and Online Resources 2, 501 matching peptides and a sequence coverage of 13.88% were detected, suggesting that the protein extracted is mouse-derived titin protein. After the identification, proteins from the left ventricle myocardium from three groups of mice were extracted and titin isoforms were separated by 2% agarose strengthened SDS-PAGE gels. As is demonstrated in the present study (Fig. 3a, b), the ratio of N2BA to N2B was significantly decreased in TAC mice as compared with sham mice. Notably, this decreased ratio of N2BA to N2B was restored by the supplementation of APN.

### Extracellular Matrix Network Reorganization

Protein expression of collagen type I was increased in TAC mice vs. sham mice, while myocardial protein expression of collagen type III was similar among three groups (Fig. 3c, d, f, g). With the treatment of APN, collagen type I expression was ameliorated, resulting in a decreased ratio of collagen type I to collagen type III (Fig. 3h). Myocardial protein expression of lysyl oxidase (Lox) was evidently increased in TAC mice compared with sham mice, which was also downregulated in APN-treated mice (Fig. 3e, i).

### Myocardial Oxidative Stress

**Fig. 4:**
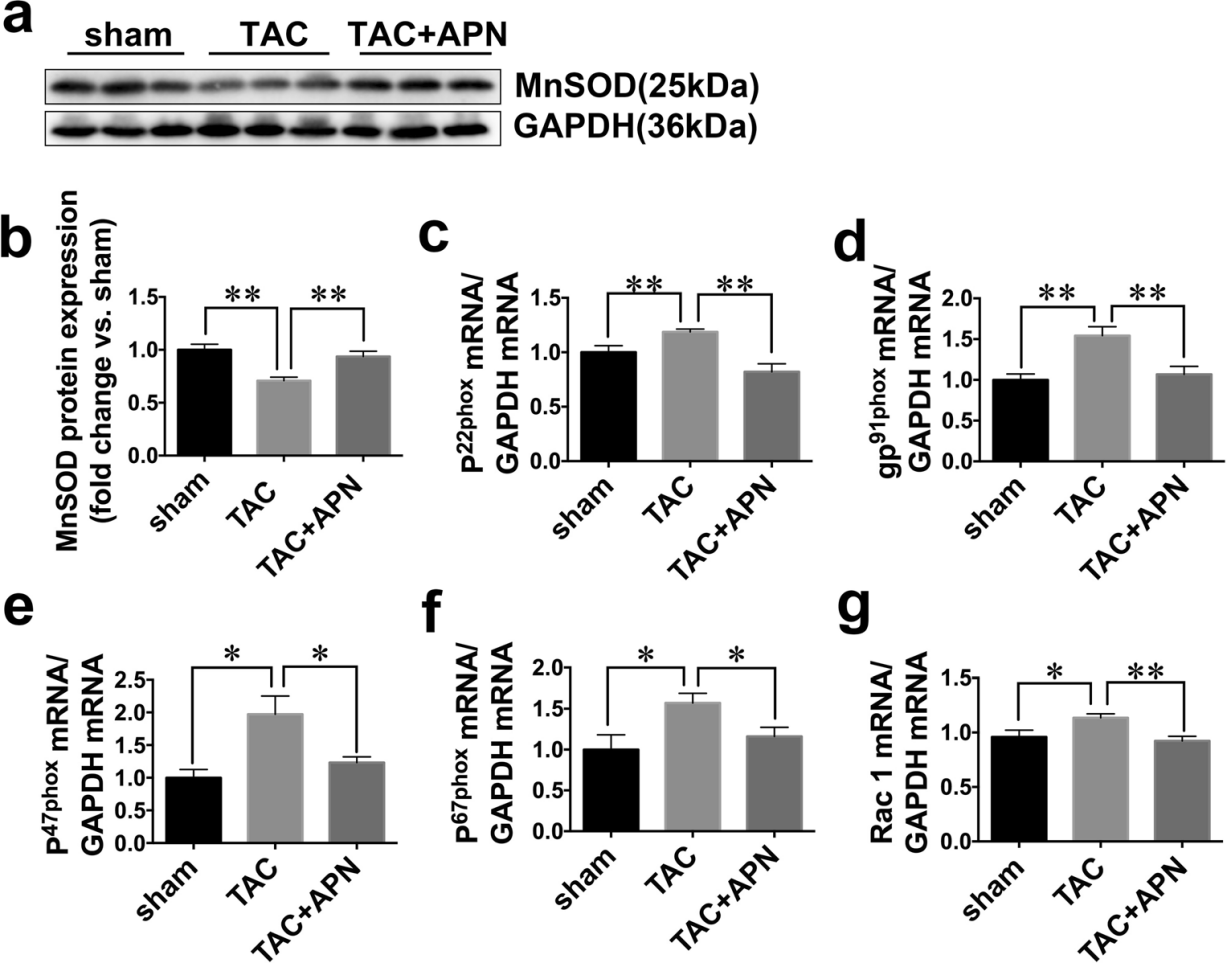
APN abated gene expressions of NADPH oxidase subunits in TAC mice. **a**, **b** Representative protein blots of MnSOD and GAPDH in mice ventricular myocardium. Values represent the mean ± SEM, *n* = 6 per group. **P < 0.01. **c–g** Relative mRNA expressions of p^22phox^, gp^91phox^, p^47phox^, p^67phox^, and Rac1. Values represent the mean ± SEM, *n* = 6 per group. **P < 0.01; *P < 0.05

The anti-oxidative protein expressions of manganese containing superoxide dismutase (MnSOD) were downregulated in TAC mice, which were restored by the treatment of APN (Fig. 4a, b). Expressions of mRNAs for the p^22phox^ and gp^91phox^ membrane components and for the p^47phox^, p^67phox^, Rac1 cytosolic components of NADPH oxidase in LV of TAC mice were increased compared with those of sham mice, which were in turn all downregulated with the treatment of APN (Fig. 4c–g).

### AMPK Was Activated by APN in TAC Mice

**Fig. 5:**
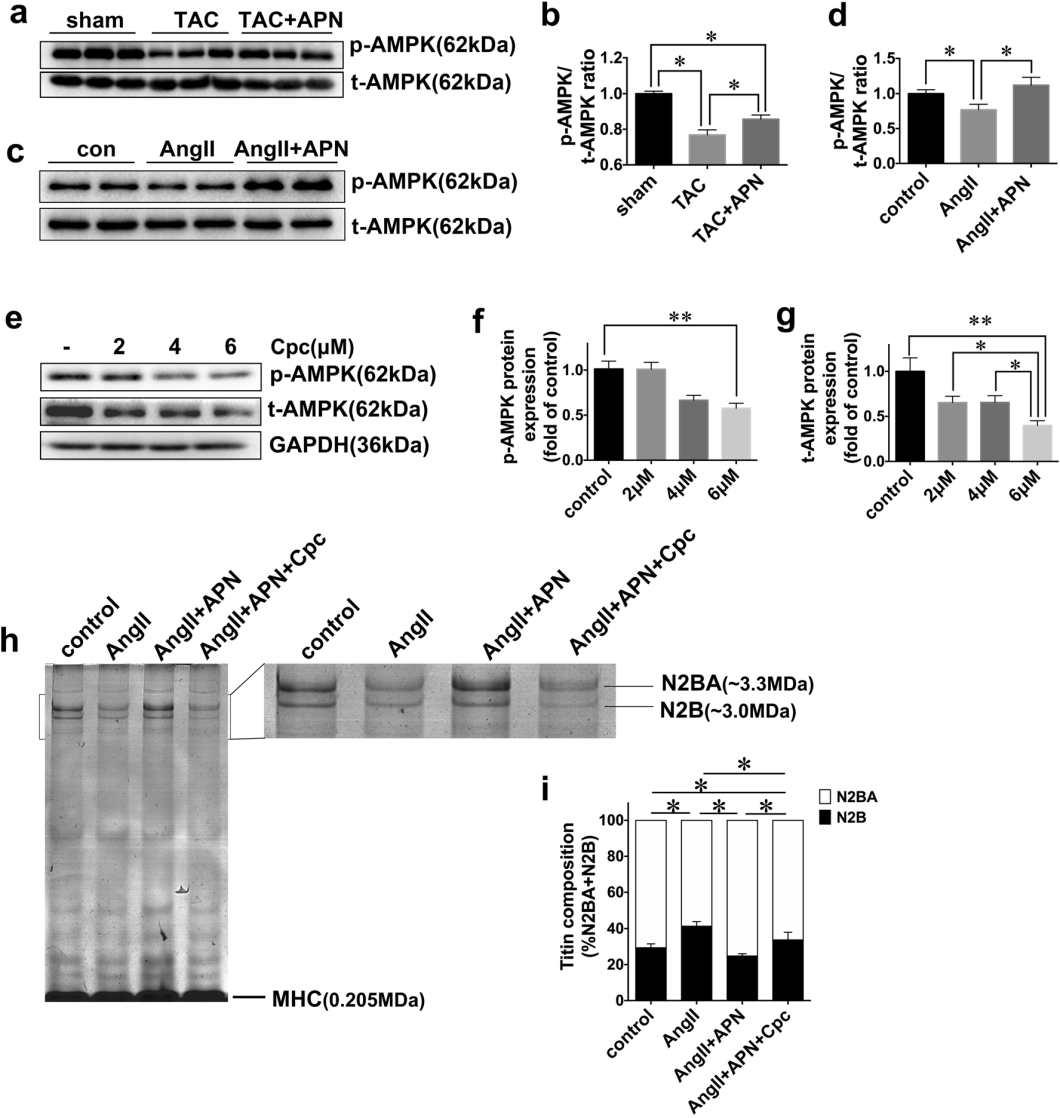
AMPK inhibition abrogated effects of APN in regulating titin isoform transformation. **a** Representative protein expressions of phosphorylated AMPK, total AMPK in mice ventricular myocardium. **b** Quantitative analysis. Values represent the mean ± SEM, *n* = 6 per group. *P < 0.05. **c** Representative protein expressions of phosphorylated AMPK, total AMPK in cardiomyocytes. **d** Quantitative analysis. Values represent the mean ± SEM. Experiments were repeated five times with cells obtained from three independent cultures. *P < 0.05. **e**–**g** Cells were under stimulation of compound C at the concentration of 2, 4, and 6 μM, respectively. *P < 0.05; **P < 0.01. **h** Representative images of titin isoforms in NRCMs treated with AngII, in the presence or absence of APN or Cpc. **i** Quantitative analysis of titin isoforms. *P < 0.05. Values represent the mean ± SEM. Experiments were repeated five times with cells obtained from three independent cultures

AMPK is a well-documented major downstream signal molecular of APN, which mediated multiple functions of APN including anti-hypertrophy [25] and anti-fibrosis [27] effects. As is shown in the present study (Fig. 5a, b), the phosphorylated AMPK was decreased in TAC mice, which was then upregulated by the treatment of APN. This implicated that AMPK may possibly be involved in the protective role of APN against diastolic dysfunction in this mice model.

### AMPK Inhibition Mitigated the Effect of APN in Titin Isoform Transformation

To explore molecular mechanism(s) behind the protective effects of APN in vitro, neonatal rat cardiomyocytes were stimulated with AngII. In line with what was observed in the myocardium of mice, phosphorylated AMPK was also elevated at the presence of APN, as compared with cells underwent AngII stimulation alone (Fig. 5c, d). Compound C at the concentration of 6 μM was utilized to inhibit AMPK (Fig. 5e, f, g). Compared with control group, AngII stimulation reduced N2BA/N2B ratio, which was increased in APN-treated group (Fig. 5f, g). With the inhibition of AMPK, N2BA/N2B ratio was reduced in APN-treated group, suggesting that the effect of APN in titin isoform modification was mediated by AMPK.

### AMPK Inhibition Abrogated Anti-hypertrophic Effect of APN

**Fig. 6:**
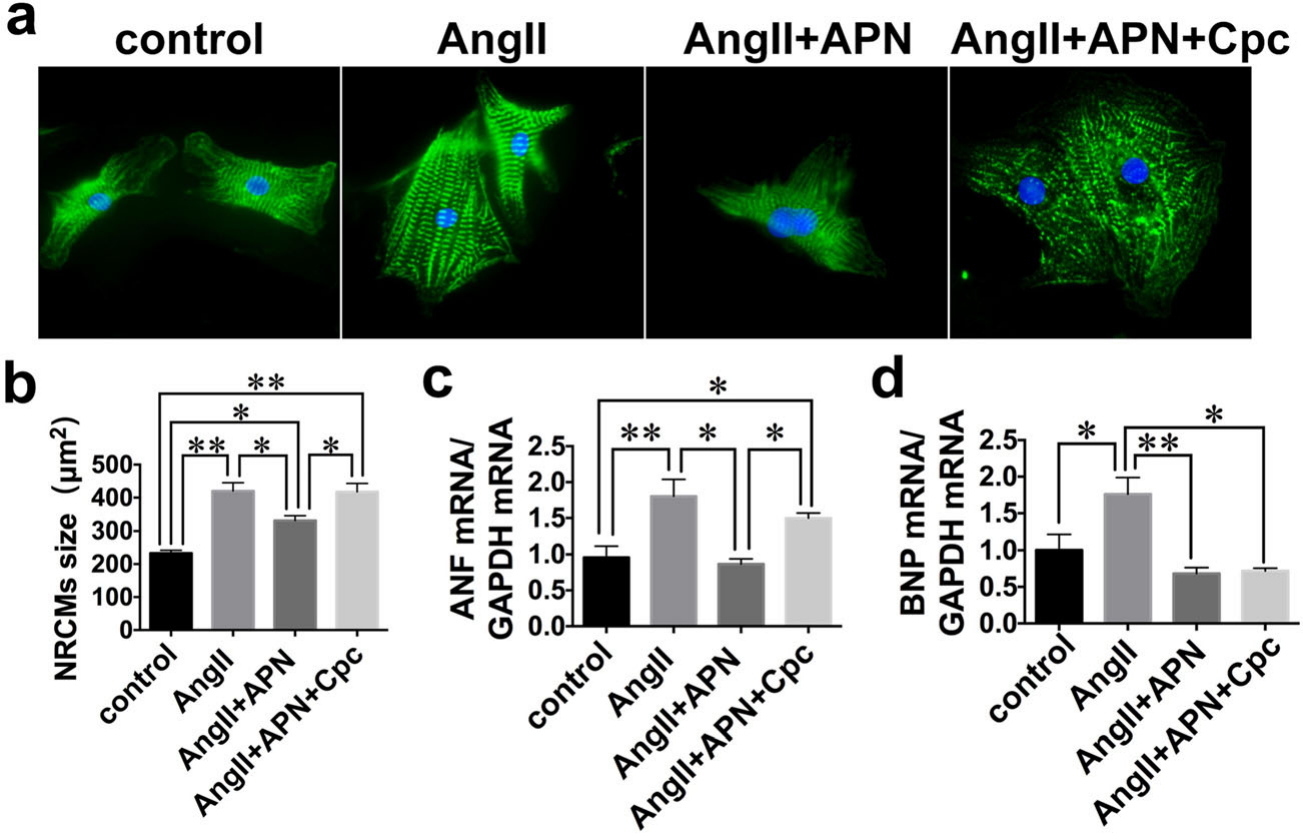
AMPK inhibition abrogated the anti-hypertrophic effect of APN. **a** Representative images of NRCMs under indicated stimulations. **b** Quantitative analysis of NRCMs size (original magnification, × 400). **c**, **d** Hypertrophic gene expressions of ANF and BNP. Values represent the mean ± SEM. Experiments were repeated five times with cells obtained from three independent cultures. *P < 0.05; **P < 0.01

AngII stimulation increased the size of cardiomyocytes as compared with control group. Cardiomyocyte size was smaller in APN-treated group vs. AngII-stimulated group, whereas this effect was disappeared with additional treatment of Cpc (Fig. 6a, b). AngII induced upregulated ANF mRNA expressions compared with control group. Treatment with APN downregulated the mRNA expression of ANF, which was in turn abrogated by Cpc. mRNA expression of BNP was also increased by the stimulation and was reduced by the treatment of APN. However, this effect was not affected by cotreatment with Cpc (Fig. 6c, d). These data indicated that AMPK mediated the anti-hypertrophic effect of APN.

## Discussion

The relationship between circulating adiponectin level and cardiovascular diseases varies according to different types of diseases. Clinical observations demonstrated an increased plasma adiponectin level among patients with reduced cardiac function and were related to increased mortality [28, 29]. This phenomenon is reported to be cause by the downregulation and (or) the phosphorylation of APN receptors, leading to increased circulation APN level and reduced biological response of targets to APN [30]. Nonetheless, clinical studies also observed that low APN level was associated with higher odds of indices of diastolic dysfunction [31, 32], implying that low adiponectin level could be a potential risk factor of cardiovascular diseases.

In the present study, we aimed to investigate the potent role of APN on pressure overload–induced mice following TAC. Our data showed that mice underwent TAC developed cardiac diastolic dysfunction and LVH [15, 17] and for the first time demonstrated that: (1) APN improved active relaxation on a cellular level by increasing the maximal re-lengthening rate and reducing time to 90% re-lengthening of cardiomyocytes in TAC mice; (2) APN exerted positive impacts on myocardial passive stiffness by upregulating titin isoform N2BA/N2B ratios within cardiomyocytes and downregulating collagen I expression, collagen I/III ratios, and Lox expression in extracellular matrix level. Moreover, APN attenuated oxidative stress, which contributed to the benefit effects of APN on the prevention of diastolic dysfunction. Furthermore, we unraveled the molecular mechanisms of APN based on in vitro experiments and demonstrated for the first time that adiponectin modulated titin isoform switching by activating AMPK signaling pathway.

Transmitral flow spectrum and tissue Doppler imaging (TDI) were performed to jointly evaluate diastolic function, since it can be defined by no one single measurement. In TAC mice, *E* velocities were increased, leading to further increased *E/A*, indicative of impaired LV compliance. Combining transmitral flow velocity with annular velocity (*E*/*E*′) has been proposed as a tool for assessing LV filling pressures that combines the influence of transmitral driving pressure and myocardial relaxation, and is reported to be highly correlated with diastolic parameters obtained by PV-loop measurements [33, 34]. In our study, *E*′ velocities were decreased in TAC mice, resulting in increased *E*/*E*′, suggesting elevated LV filling pressures and abnormal myocardial relaxation caused by TAC. These parameters were downregulated by the administration of APN for 2 weeks. DT and IVRT tend to decrease in TAC mice. This may be explained by the biphasic response of DT and IVRT to increasing diastolic dysfunction, with DT and IVRT prolonged in patients in an early stage of diastolic dysfunction [34], “normalized” in further progression of ventricular diastolic dysfunction, and shortened in end-stage disease with increased LV filling pressures, producing a “restrictive” transmitral pattern [35]. This may also justify the disparities in DT and IVRT of different mice models presented with diastolic dysfunction [15, 16, 36, 37].

Diastolic function is partly comprised of the active process of pressure decay (relaxation) during early diastole [6]. It is reported that 80% of patients with diastolic dysfunction also show signs of impaired LV relaxation [3]. Anything that interferes with cross-bridge detachment or with preceding calcium removal from the cytosol has the potential to delay relaxation. Alterations in myocyte calcium handling proteins, including the sarcoplasmic reticular Ca^2+^-ATPase (SERCA2a) and its modulator PLB, have been implicated in altering the calcium transient in failing hearts and contributing to delayed relaxation [38]. Previous studies [14] showed that APN attenuated diastolic dysfunction in aldosterone-infused mice by downregulating PKA-dependent PLB phosphorylation at Ser16 and CaMKII-dependent PLB phosphorylation at Thr17, implying that APN may attenuate aldosterone-induced diastolic dysfunction through regulating calcium handling protein–related cardiomyocytes active relaxation. In this study, we isolated cardiomyocytes from three groups of mice and test the active relaxation of cardiomyocytes directly. We presented evidence for delayed active relaxation in single myocytes from diastolic dysfunction hearts induced by TAC, as reflected by increased maximal rates of cell re-lengthening, prolonged time-to-50% re-lengthening and time to 90% re-lengthening. Addition of APN corrected these abnormalities except time-to-50% re-lengthening. Thus, our data suggested that APN exerted positive impacts on TAC-induced diastolic dysfunction by improving active relaxation of single myocytes, which might be associated with the effect of APN on regulating calcium handling proteins PLB phosphorylation as observed in aldosterone-induced mice.

Another important component of diastolic function is the passive stiffness, specifically governed by titin compliance–based cardiomyocyte stiffness and extracellular matrix–based stiffness [6, 39]. Titin stiffness is mainly defined by the sarcomeric composition of 2 main cardiac titin isoforms N2BA (3.2~3.3 MDa, long and compliant) and N2B (3.0 MDa, shorter and stiffer) [40]. van Heerebeek et al. [41] observed higher expression of N2B titin isoform in myocardium from HFpEF patients, which was speculated as being responsible for the observed higher cardiomyocyte passive stiffness. Experimentally inhibiting RNA binding motif-20 (RBM-20), a splicing factor that manipulates titin isoform shift towards N2BA isoform, resulted in attenuated diastolic dysfunction induced by pressure overload [23]. In line with above findings, we detected a significant decrease in N2BA/N2B ratio from the LV of TAC mice. Treatment of APN for 2 weeks profoundly abated N2B expression and restored the N2BA/N2B ratio. Collagen type I predominates in heart fibrillar collagens with approximately 85%, forms large, well-structured fibbers, whereas type III collagen represents 11% of the total collagen protein in the heart, typically forms a fine reticular network [42] [43]. A small increase in the concentration of collagen type I contributes profoundly to myocardial stiffness. Collagen fibrils are covalently linked to one another by the process of cross-linking, resulting in insoluble fibers with increased material stiffness and insusceptible to degradation by matrix metalloproteinases (MMPs). In hypertensive patients with heart failure, Lox-mediated collagen cross-linking facilitates the LV passive stiffness to increase, resulting in elevated LV filling pressure [7]. Here, we observed increased protein expression in collagen type I and in collagen type I/III ratios, as well as increased protein expression of Lox in the myocardium of TAC mice, indicating aggravated ECM might be related to the enhanced myocardial stiffness in hearts of TAC mice. Importantly, these abnormalities were amended by APN treatment. Notably, collagen type III expression seemed unchanged among three groups. This may due to its low expression level in extracellular matrix. Moreover, in models of hypertensive cardiac fibrosis, type I collagen exhibits more intense and prolonged upregulation than collagen III [44, 45]. Collectively, these data suggested that APN alleviated myocardial stiffness by maintaining titin compliance in cardiomyocytes and reducing extracellular matrix deposition, leading to improved diastolic function in TAC-induced mice.

Oxidative stress is not only reported to induce cardiac hypertrophy [46] and myocardial fibrosis [47], but also to increase titin-based passive stiffness [48, 49] via promoting the formation of disulfide bridges within the disordered N2-Bus element of cardiac titin, thus playing an important role in pathological progression of diastolic dysfunction. As previously reported, APN protected hearts against ischemia/reperfusion injury by inhibition of iNOS and nicotinamide adenine dinucleotide phosphate (NADPH)-oxidase protein-gp^91phox^ expression and resultant oxidative/nitrative stress. In the present study, APN was observed to increase protein expressions of MnSOD, an antioxidant enzyme important for protection against oxidative stress [50]. In addition, APN treatment attenuated NADPH oxidase gene expressions in myocardium from TAC mice. These data implicated that the inhibition of oxidative stress by APN may be associated with improved cardiac remodeling and decreased titin-based myocardial stiffness, contributing to improved diastolic function in TAC-induced mice.

AMPK is a stress-activated protein kinase that participates in the regulation of energy and metabolic homeostasis [51]. AMPK activation is related to multiple beneficial effects on the heart including facilitating glucose and fatty acid uptake, inhibiting protein synthesis and hypertrophic responses [52]. While some studies showed that the roles of APN in suppressing glucose-induced ROS in endothelial cells [53] and activating of ceramidase activity [54] were performed independently of AMPK, many others reported that APN functions in an AMPK-dependent manner, such as attenuating cardiac hypertrophy [25] and ischemia/reperfusion injury [55]. In the present study, consistent with previous findings [56, 57], we detected a decrease in the protein expression of phosphorylated AMPK in TAC-induced mice. By the treatment of APN, this was reversed. Angiotensin II (AngII) promotes cell growth, proliferation, migration, oxidative stress, and implicated in inflammation, all processes which contribute to remodeling of the heart and vascular, ultimately leading to the development and progression of various cardiovascular diseases, including heart failure [58]. In in vitro study, AngII stimulation was applied to partially mimic the pathological mechanism of cardiac myocytes in hearts of TAC mice. In line with above findings, the upregulation of phosphorylated AMPK was also seen in APN-treated NRCMs under the stimulation of AngII. The abundance of N2BA titin in adult hearts is very low. In neonates, expression of N2BA titin was higher in rats than in mice [59]. To explore the molecular mechanisms in the observed effects of APN in titin isoform transformation, we performed in vitro experiment using NRCMs. Our data revealed that treatment of APN increased N2BA/N2B ratio in NRCMs under the stimulation of AngII. Moreover, addition of APN decreased the size of NRCMs and hypertrophic gene expressions of ANF, BNP. Nonetheless, these effects of APN were abrogated by the inhibition AMPK. These data demonstrated that AMPK mediated effects of APN on modulating titin isoform swift and inhibition cardiac hypertrophy, suggesting that the protective role of APN against diastolic dysfunction induced by TAC might be mediated, at least partially, by AMPK signaling pathway.

### Study Limitations

First, further in vivo study based on AMPK knock-out mice is warranted to confirm the precise molecular mechanism(s). However, this in vivo experiment in our laboratory is hampered due to technical issues. We will consider to perform related experiments in the follow-up research. Second, while the present study focused on protective role of APN on TAC mice after 4 weeks, its effect on a longer term post-TAC is another important topic to be further investigated. Third, the “sham+APN” group should be included in future studies to observe the effect of APN on sham mice. Finally, APN that released by adipocyte and cardiac myocytes may change as reactions to TAC, this observation is not done in our study. This would be a topic of another study of our group.

### Conclusion

Collectively, APN supplementation attenuated diastolic dysfunction induced by TAC, which is associated with regulating cardiomyocytes active relaxation, passive stiffness. The beneficial effects of APN in this mice model might be partly mediated by AMPK signaling pathway.

## Electronic Supplementary Material

Online Resource 1 (PDF 441 kb)

Online Resource 2 (PDF 24644 kb)

## Abbreviations

APN: Adiponectin;
*A*: Peak late transmitral flow velocity;
BW: Body weight;
DT: Early filling deceleration time;
*E*: Peak early transmitral flow velocity;
*E*′: Peak early diastolic myocardial velocity;
FS: Fractional shortening;
HR: Heart rate;
HW: Heart weight;
HW/BW: Heart weight to body weight ratio;
HW/TL: Heart weight to tibia length ratio;
IVRT: Isovolumetric relaxation time;
Lox: Lysyl oxidase;
LV: Left ventricular;
LVH: Left ventricular hypertrophy;
LVPW, s: Systolic left ventricular posterior wall;
LVPW, d: Diastolic left ventricular posterior wall;
LVID, d: Diastolic left ventricular internal dimension;
LVID, s: Systolic left ventricular internal dimension;
LVEF: Left ventricular ejection fraction;
TAC: Transverse aorta constriction;
TL: Tibia length;
