# Supplementary material for: Effects of Adiponectin on Diastolic Function in Mice Underwent Transverse Aorta Constriction
Source: J Cardiovasc Transl Res. 2019 Oct 16;13(2):225–37. doi: 10.1007/s12265-019-09913-1 (PMC7166206; doi:10.1007/s12265-019-09913-1)
Supplement: Supplementary file 1 — (PDF 441 kb) [file 12265_2019_9913_MOESM1_ESM.pdf]

Journal of Cardiovascular Translational Research

Effects of adiponectin on diastolic function in mice underwent transverse aorta constriction

Xueting Han, M.D., Ph.D. [1*], Yanyan Wang, M.D., Ph.D.[1*], Mingqiang Fu, M.D., Ph.D.[1*], Yu Song, M.D., Ph.D.[1], Jingfeng Wang, M.D., Ph.D.[1], Xiaotong Cui, M.D., Ph.D.[1], Yuyuan Fan, M.D.[2], Juan Cao, M.D. [2], Jie Luo, M.D. [2], Aijun Sun, M.D., Ph.D. [1], Yunzeng Zou, M.D., Ph.D.[1], Kai Hu, M.D., Ph.D.[1], Jingmin Zhou, M.D., Ph.D.[1#], Junbo Ge, M.D., Ph.D.[1#]

[1]*Department of Cardiology, Shanghai Institute of Cardiovascular Diseases, Zhongshan Hospital, Fudan University, Shanghai, China*

[2]*North Sichuan medical College, Nanchong, Sichuan, China*

** These authors contributed equally to this work.*

# Correspondence to: Professor Jingmin Zhou, Professor Junbo Ge

Tel.: +86-21-64041990;

E-mail: zhou.jingmin@zs-hospital.sh.cn (Jingmin. Zhou), jbge@zs-hospital.sh.cn (Junbo. Ge).

SUPPLEMENTAL MATERIALS

1) Supplemental Methods

2) Supplemental Tables

3) Supplemental Figures and Figure Captions

4) Supplemental References

1) Supplemental Methods

Adult Cardiac Myocytes Isolation. Mouse myocytes from the left ventricle were isolated via enzymatic dissociation, as described earlier [1]. 4 weeks after intervention, mice were anesthetized and the chest was opened to expose the heart. Descending aorta was cut, and the heart was immediately flushed by injection into the right ventricle of 7 mL EDTA buffer containing (mmol/L) NaCl 130, KCl 5, NaH_2PO_4 0.5, HEPES 10, Glucose 10, BDM 10, Taurine 10, EDTA 5, pH 7.8. Ascending aorta was clamped using forceps, and the heart was transferred to a 60-mm dish containing fresh EDTA buffer. Digestion was achieved by sequential injection into the left ventricle (LV) of 10 mL EDTA buffer, 3 mL perfusion buffer (containing (mmol/L) NaCl 130, KCl 5, NaH_2PO_4 0.5, HEPES 10, Glucose 10, BDM 10, Taurine 10, $MgCl_2$ 1, pH 7.8), and 50 mL collagenase buffer (containing 0.5 mg/ml Collagenase 2 (Worthington, USA), 0.5 mg/ml Collagenase 4 (Worthington, USA), 0.05mg/ml Protease XIV (Sigma-Aldrich, Singapore)). LV was then separated and gently pulled into 1-mm^3 pieces using forceps. Cellular dissociation was completed by gentle trituration, and enzyme activity was inhibited by addition of 5 mL stop buffer (containing (mmol/L) NaCl 130, KCl 5, NaH_2PO_4 0.5, HEPES 10, Glucose 10, BDM 10, Taurine 10, $MgCl_2$ 1 and 5% sterile fetal bovine serum, pH 7.8). Cell suspension was passed through a 100 µm cell strainer (falcon, USA). The calcium concentration was restored gradually to physiological level using 3 intermediate calcium reintroduction buffers. A yield of 80%~90% rod-shaped cardiac myocytes with clear sarcomere striations were achieved.

Titin Isoform Separation. Flash-frozen LV tissue were prepared as previously described [2-4]. Myocardial samples were homogenized

between glass pestles cooled in liquid nitrogen. Tissues were primed at -20°C for a minimum of 20 min and were solubilized in sample buffer containing 8M urea, 2M thiourea, 3% SDS, 75mM DTT, 10% glycerol, 0.03% bromophenol blue, 0.05M Tris-HCl pH 6.8 and protease inhibitors ((in mmol/L) E64 0.04, Leupetin 0.16, and PMSF 0.2) at 60°C for 10 min. Samples were centrifuged for 5 min at 13000 rpm, aliquoted, flash frozen in liquid nitrogen and stored at -80°C. Titin isoform separation was performed as previously described [5,6]. The solubilized samples were electrophoresed on a 2% agarose strengthened sodium dodecyl sulfate polyacrylamide (SDS-PAGE) gel. Each gel was run at 2 mA constant current for at least 12hrs or overnight. Then, gels were stained using Coomassie brilliant blue for 2hrs at room temperature, washed with 20% ethanol and 20% acetic acid and scanned using a commercial scanner (Epson Image Scanner III, Epson Corporation, Long Beach CA). Band densitometry was analyzed by NIH Image J (1.51e) software.

Titin identification by Nano-HPLC-MS/MS analysis

In-gel Digestion. Protein slice was excised and plated into a 96-well microtitre plate. Excised slices were firstly destained twice with 200 μl of 50 mM NH_4HCO_3 and 50% acetonitrile and then dried twice with 200μl of acetonitrile. Afterwards, the dried pieces of gels were incubated in ice-cold digestion solution (trypsin 12.5 ng/μl and 20mM NH_4HCO_3) for 20 min and then transferred into a 37°C incubator for digestion overnight. Finally, peptides in the supernatant were collected after extraction twice with 200μl extract solution (5% formic acid in 50% acetonitrile). The peptide solution described above was dried under the protection of N_2.

Nano-HPLC-MS/MS analysis and database Searching. The peptides were resuspended with 0.1% formic acid, separated by Nano Aquity UPLC system and analyzed by on-line electrospray tandem mass spectrometry(Q-

Exactive). Tandem mass spectra were extracted by Proteome Discoverer software (Thermo Fisher Scientific, version 1.4.0.288) and analyzed using Mascot (Matrix Science, London, UK; version 2.3). The search parameters were as follows: Swissprot Mouse database, trypsin digest with two missing cleavage, MS tolerance was set at 10 ppm, MS/MS tolerance of 0.05Da, fixed modifications of iodoacetamide at Cys, variable modifications of oxidation at Met.

2) Supplemental Tables

Table S1 Echocardiographic parameters of mice after 2 weeks of TAC.

**At 2wk After Surgery**	**sham**	**TAC**
**LV Structure**		
**LVPW, d, mm**	0.54±0.03	0.72±0.04*
**LVPW, s, mm**	0.79±0.08	1.04±0.03**
**LVID, d, mm**	3.56±0.08	3.49±0.12
**LVID, s, mm**	2.49±0.12	2.37±0.14*
**Diastolic Function**		
**E, mm/s**	516.50±5.24	611.10±26.51**
**A, mm/s**	359.4±5.855	360.50±18.56
**E/A**	1.46±0.04	1.86±0.07**
**E’, mm/s**	22.80±1.06	17.89±0.59**
**E/E’**	28.65±1.19	38.44±0.46**
**IVRT**	19.70±1.84	18.06±2.247
**DT**	20.86±1.69	18.92±3.315
**Systolic Function**		
**LVEF, %**	67.19±2.523	68.57±2.60
**FS, %**	35.38±1.10	35.34±2.08

LVPW, s, systolic left ventricular posterior wall; LVPW, d, diastolic left ventricular posterior wall; LVID, d, diastolic left ventricular internal dimension; LVID, s, systolic left ventricular internal dimension; E, peak

early transmitral flow velocity; A, peak late transmitral flow velocity; E’, peak early diastolic myocardial velocity; IVRT, isovolumetric relaxation time; DT, early filling deceleration time; LVEF, left ventricular ejection fraction; FS, fractional shortening. Values represent the mean ± SEM, n=8-10 per group. **, P< 0.01 vs. sham; *, P< 0.05 vs. sham

3) Supplemental Figures and Figure Captions

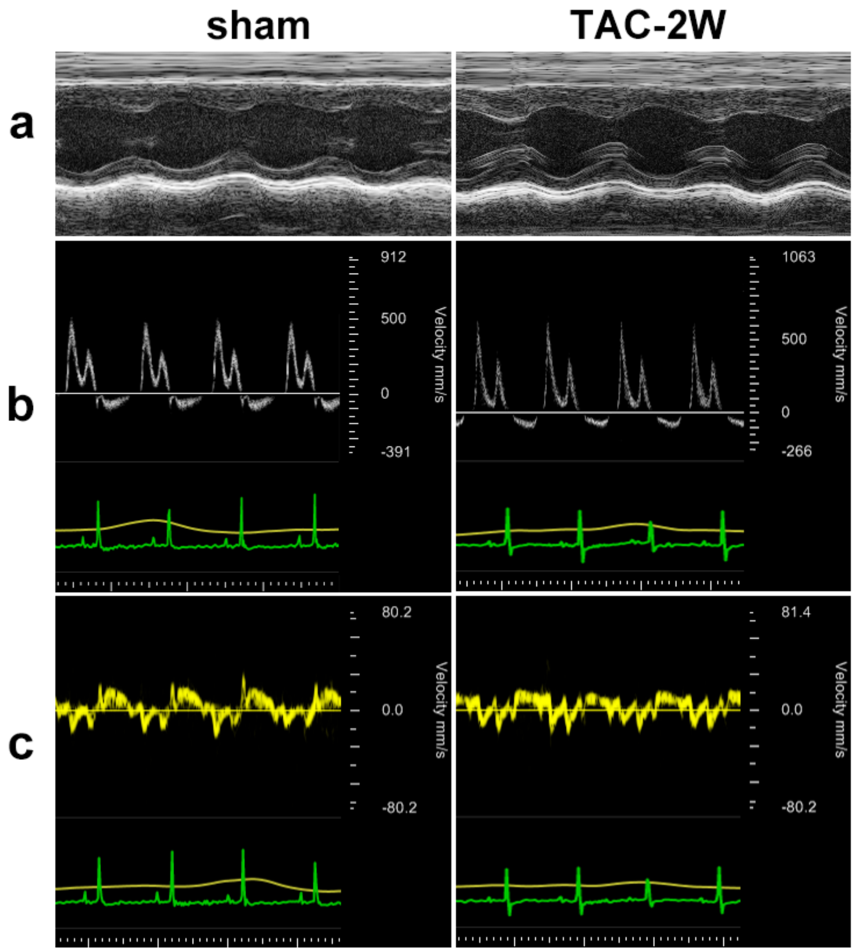

Fig. S1 Echocardiography measurements of mice after 2 weeks of TAC. **a** M-mode echocardiography showed increased left ventricular posterior wall thickness (LVPW) in TAC mice. **b** Mitral inflow pattern and **c** mitral annular velocity of TAC group revealed progressive diastolic dysfunction

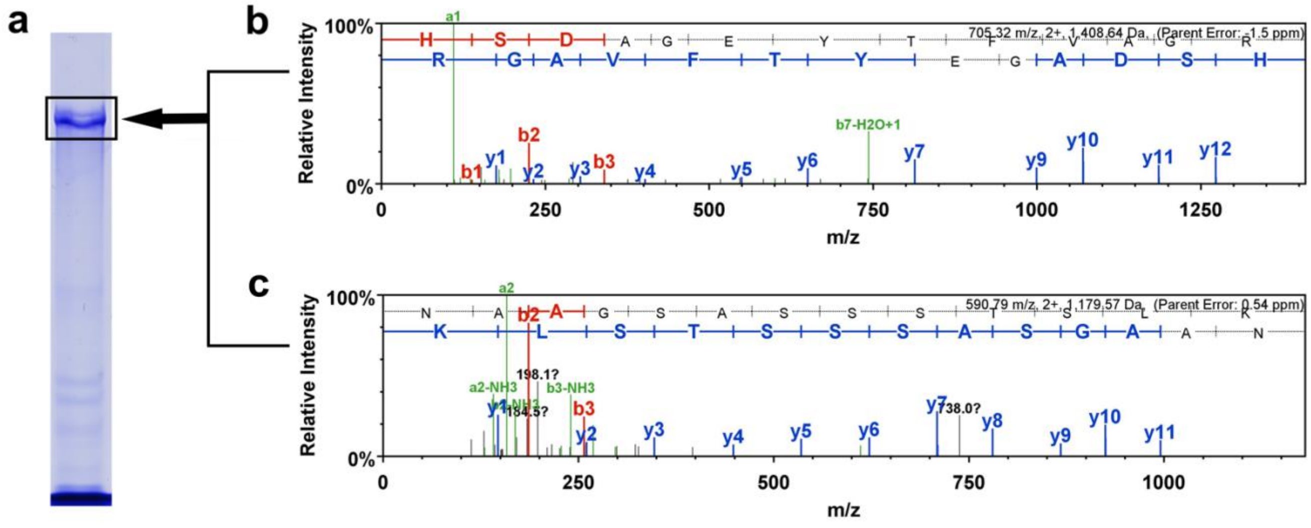

Fig. S2 Separation of titin by 2% agarose strengthened SDS-PAGE gels and Nano-HPLC-MS/MS analysis of titin from mouse heart of left ventricle. **a** Titin protein bands in a 2% agarose strengthened SDS-PAGE gel; Proteins were extracted from mice left ventricular myocardium. The frame part was sliced and subjected to Nano-HPLC-MS/MS analysis. **b** Representative tandem mass spectrum of peptide from titin protein. The amino acid sequence is HSDAGEYTFVAGR. The iron score is 83; **c** Representative tandem mass spectrum of peptide from titin protein. The amino acid sequence is NAAGSASSSTSLK. The iron score is 98

4) Supplemental References

1. Ackers, J. M., Li P.Y., Holmes, A.P., O'Brien, S.M., Pavlovic, D., & Foo, R.S. (2016). A simplified, langendorff-free method for concomitant isolation of viable cardiac myocytes and nonmyocytes from the adult mouse heart. Circ Res, 119(8), 909-920.
2. Warren, C.M., Krzesinski, P.R., & Greaser, M.L. (2003). Vertical agarose gel electrophoresis and electroblotting of high-molecular-weight proteins. Electrophoresis, 24(11), 1695-1702.
3. Methawasin, M., Strom, J.G., Slater, R.E., Fernandez, V., Saripalli, C., & Granzier, H. (2016). Experimentally increasing the compliance of titin through RNA binding motif-20 (RBM20) inhibition improves diastolic function in a mouse model of heart failure with preserved ejection fraction. Circulation, 134(15), 1085-1099.
4. Zhu, C., Yin, Z., Tan, B., & Guo, W. (2017). Insulin regulates titin pre-mrna splicing through the PI3k-Akt-mTOR kinase axis in a RBM20-dependent manner. Biochim Biophys Acta, 1863(9), 2363-2371.
5. Kotter, S., Kazmierowska, M., Andresen, C., Bottermann, K., Grandoch, M., Gorressen, S., et al. (2016). Titin-based cardiac myocyte stiffening contributes to early adaptive ventricular remodeling after myocardial infarction. Circ Res, 119(9), 1017-1029.
6. Hamdani, N., Franssen, C., Lourenco, A., Falcao, P.I., Fontoura, D., Leite, S., et al. (2013). Myocardial titin hypophosphorylation importantly contributes to heart failure with preserved ejection fraction in a rat metabolic risk model. Circ Heart Fail, (6)6, 1239-1249.
