# Supplementary material for: Effects of Adiponectin on Diastolic Function in Mice Underwent Transverse Aorta Constriction
Source: J Cardiovasc Transl Res. 2019 Oct 16;13(2):225–37. doi: 10.1007/s12265-019-09913-1 (PMC7166206; doi:10.1007/s12265-019-09913-1)
Supplement: Supplementary file 2 — (PDF 24644 kb) [file 12265_2019_9913_MOESM2_ESM.pdf]

TITIN_MOUSE (100%), 3,906,107.2 Da
Titin OS=Mus musculus GN=Ttn PE=1 SV=1
11 exclusive unique peptides, 13 exclusive unique spectra, 768 total spectra, 4191/35213 amino acids (12% coverage)

```
MTTQAPMFTQ PLQSVVVLEG STATFEAHVS GSPVPEVSWF RDGQVISTST LPGVQISFSD GRARLMIPAV TKANSGRYSL
RATNGSGQAT STAELLVTAE TAPPNFSQRL QSMTVRQGSQ VRLQVRVTGI PTPVVKFYRD GAEIQSSLDF QISQEGDLYS
LLIAEAYPED SGTYSVNATN SVGRATSTAE LVVQGEEVVP AKKTKTIVST AQISETRQTR IEKKIEAHFD ARSIATVEMV
IDGATGQLPH KTPPRIPPKP KSRSPTPPSI AAKAQLARQQ SPSPIRHSPS PVRHVRAPTP SPVRSVSPAG RISTSPIRSV
KSPLLIRKTQ TTTMATGPEV PPPWKQEGYV ASSTEAEMRE TTMTSSTQIR REERWEGRYG VQEQVTISGA AAAAASASVS
SSFTAGAITT GTKEVKQETD KSAAVATVVA AVDMARVREP AISAVEQTAQ RTTTTAVHIQ PAQEQARKEA EKTAVTKVVV
AADKAKEQEL KSRTREVMVT TQEQTHISHE QIRKETEKAF VPKVVISATK AKEQETRITG EITTKQEQKR ITQETIRQET
EEIAASMVVV ATAKSTKLEA AVGVQEETAI QQDQMHLTHE QMMKETRKTV VPKVIVATPK IKEQDLVSRS REAITTKRDQ
VQITQEKKRK EVETTALSTI AVATAKAKEQ ETVLRSREAM ATRQEHIQVT HGQVGVGKKA EAVATVVAAV DQARVREPRE
PTHVEESHSQ QTTLEYGYKE HISTTKVPEQ PRRPASEPHV VPQAVKPAVI QAPSETHIKT TDQMGMHISS QVKKTTDIST
ERLVHVDKRP RTASPHFTVS KISVPKTEHG YEASIAGSAI ATLQKELSAT SSTQKITKSV KAPTVKPGET RVRAEPTPSP
QFPFADMPPP DTYKSQAGIE VKKEVGVSIS GSTVREEHFE VLRGREAKVT ETARVPAPAE VPVTPPTLVS GLKNVTVIEG
ESVTLECHIS GYPSPKVTWY REDYQIESSI DFQITFQGGI ARLMIREAFA EDSGRFTCSA VNEAGTVSTS CYLAVQVSEE
FDKETTLTEK FATEEKRFVE SRDVVMTDTS ITEEQAGPGE PAAPFFISKP VVQKLVEGGS VVFECQIGGN PKPHVYWKKS
GVPLTTGYRY KVSYNKQTGE CRLVISMTFA DDAGEYTIVI RNKHGETSAS ASLLEEADYE ALVKTQQEML YQTQMSTFIQ
EPKVGEIAPG FAYSEYEKEY EKEQALIRKK MAKDTVMVRT FVEDQEFHIS SFEERLIKEI EYRIIKTTLE ELLEEDGEEK
MAVDISESEA IESGFDIRIK NYRILEGMGV TFHCKMSGYP LPKIAWYKDG KRIRHGERYQ MDFLQDGRAS LRIPVVLPED
EGIYTAFASN IKGNAICSGK LYVEPAAPFS APTYMPTPEA VSRIRSVSPR SLSRSPIRMS PAMSPARMSP ARMSPARMSP
ARMSPGRRLE ETDESQLERL YKPVFVLKPA SFKCLEGQTA RFDLKVVGRP MPETFWFHNG QQIVNDYTHK VVIKEDGTQS
LIIVPASPSD SGEWTVVAQN RAGKSTISVT LTVEAVEHQI KPAFVEKLKN VNIKEGSRLE MKVRATGNPN PDIVWLKNSD
IIVPHKYPRI RIEGTRGEAA LKIDSIISQD SAWYTATAIN KAGRDTTRCK VNVEVEFAEP EPERKLIIPR GTYRAKEIAA
PELEPLHLRY GQEQWEEGDL YDKEKQQKPF FKKKLTSLRL KRFGPAHFEC RLTPIGDPTM VVEWLHDGKP LEAANRLRMI
NEFGYCSLDY GAAYSRDSGV ITCRATNKYG TDHTSATLIV KDEKSLVEES QLPDGKKGLQ RIEELERMAH EGALTGVTTD
```

[illegible]
